# Einsamkeit im Pflegeheim – Erleben und Maßnahmen zur Verringerung: Eine Literaturübersicht

**DOI:** 10.1007/s00391-021-01881-z

**Published:** 2021-04-15

**Authors:** Lukas Plattner, Cornelia Brandstötter, Piret Paal

**Affiliations:** Institut für Pflegewissenschaft und -praxis, Paracelsus Medizinische Privatuniversität, Strubergasse 21, 5020 Salzburg, Österreich

**Keywords:** Einsamkeitserleben, Pflege, Stationäre Langzeitpflege, Alleinsein, Vereinsamung, Feeling lonely, Nursing, Long-term care facilities, Desolation, Aloneness

## Abstract

**Hintergrund:**

Public Health und Gesundheitsökonomie betrachten Einsamkeit als einen wichtigen Einflussfaktor auf die Gesundheit und Lebensqualität aller Menschen. Für ältere Menschen kann Einsamkeit als evtl. wichtigste Determinante der Gesundheit betrachtet werden.

**Ziel der Arbeit und Fragestellungen:**

Im Rahmen dieser Arbeit sollen das Erleben von Einsamkeit aus der Perspektive von Bewohner*innen der stationären Langzeitpflege beschrieben und Interventionen zur Verringerung von Einsamkeit durch Pflegepersonen aufgezeigt werden.

**Material und Methode:**

Systematische Literaturrecherche.

**Ergebnisse und Diskussion:**

Über die eigene Einsamkeit zu sprechen, ist für viele Bewohner*innen nicht leicht. Die Ausprägungen der Einsamkeit variieren vom Alleinsein über Langeweile bis zu dem Gefühl, sich nicht zu Hause zu fühlen. Die Maßnahmen zur Reduktion von Einsamkeit reichen über die Anwendung von Lachyoga, tiergestützter Therapie bis zu technologischen Maßnahmen oder dem Einsatz von Freiwilligen.

**Zusammenfassung:**

Als zentral und am wirkungsvollsten haben sich jene Interventionen erwiesen, welche die spirituelle Ebene des Beziehungsaufbaus und Vertrauen fokussieren.

**Zusatzmaterial online:**

Die Online-Version dieses Beitrags (10.1007/s00391-021-01881-z) enthält 2 Tabellen: die Einschluss- und Ausschlusskriterien der Literatursuche sowie die deutschen und englischen Suchbegriffe. Beitrag und Zusatzmaterial stehen Ihnen im elektronischen Volltextarchiv auf https://www.springermedizin.de/link/10.1007/10.1007/s0391-021-01881-z zur Verfügung. Sie finden die Tabellen am Beitragsende unter „Supplementary Material“

## Abstract (en)

**Background:**

Public health and health economics consider loneliness to be an important determinant of health and quality of life for all people. For older people, loneliness can be considered as possibly the most important health determinant.

**Objective:**

The purpose of this paper is to describe the experience of loneliness from the perspective of residents in residential long-term care and to identify interventions to reduce loneliness by caregivers.

**Material and methods:**

Systematic literature search and study evaluation.

**Results:**

Talking about their loneliness is not easy for many residents. The manifestations of loneliness vary from being alone, to boredom and to not feeling at home. Interventions range from the use of laughter yoga and animal-assisted therapy to technological measures and the use of volunteers.

**Conclusion:**

Measures that focus on the spiritual level of relationship building and trust have proven to be most effective.

Einsamkeit wird als ein subjektives Gefühl des Alleinseins, der Separierung oder als ein Entferntsein von anderen beschrieben [1]. Kahl führt in einem philosophischen Diskurs an, dass es sowohl eine negative Einsamkeit als auch eine positive Einsamkeit gebe. Kahl beschreibt die Einsamkeit als „(…) die Unterschiedenheit, Abgeschiedenheit, Abgesondertheit des Einzelnen von seiner Umwelt und seinen Mitmenschen, und zwar, weil er ein Einzelner ist. Einsamkeit ist nicht Vereinsamung, Verlassenheit, Verlorenheit, sondern ein existenzieller Grundtatbestand (…)“ [2]. Somit kann positive Einsamkeit als hohes Gut betrachtet werden, durch welches Menschen Erholung und Geborgenheit erfahren können. Positive Einsamkeit wird mit Seelenfrieden, innerer Stärke und selbstgesteuertem Handeln verbunden [3]. Kahl führt weiter den spirituellen Charakter der Einsamkeit an, in dem manche Menschen Trost und Zuversicht finden, andere dagegen eine Erschütterung erleben [2]. Laut Weltgesundheitsorganisation (WHO) entsteht die spirituelle Dimension „im Inneren der Menschen und innerhalb von Gemeinschaften in Übereinstimmung mit ihren sozialen und kulturellen Strukturen“ [4]. Jedoch tritt Einsamkeit häufig in der negativen Ausprägung auf und zeigt sich als unerfülltes Bedürfnis nach Intimität und sozialen Kontakten [1]. Diese Form der Einsamkeit wird etlichen Gesundheitsproblemen physischer und psychischer Art in Verbindung gebracht [5–7]. Aus der sozio- und gesundheitspolitischen Sicht lässt sich Einsamkeit als eine weltweit umfassende Pandemie erfassen [8].

## Hintergrund und Problembeschreibung

Derzeit existieren nur geringfügige Interventionen zur Reduktion der Einsamkeit, die in Pflegeheimen getestet und evaluiert wurden [9]. Jedoch steigert sich die Zahl von Publikationen, die das Thema ansprechen und nach Lösungen suchen [1, 10–15], da es sich um ein komplexes gesundheitsgefährdendes Phänomen handelt [16, 17]. Einsamkeit wird mit einer Vielzahl an negativen funktionalen, psychosozialen und physischen Konditionen in Verbindung gebracht. Dazu gehören depressive Symptome, gestörter zirkadianer Rhythmus, Reduktion mentaler und kognitiver Funktionen, Suizidalität, vermehrter Alkoholkonsum, Übergewicht und eine erhöhte Mortalität [18–20]. Als Ursache von Einsamkeit werden auch personenbedingte und heimstrukturelle Faktoren in Betracht gezogen. Hierzu zählen physische Trennung, erschwerte Kontaktaufnahme, fehlende außerinstitutionelle Kontakte sowie starre Hierarchien, Mangel an Aufgaben und Abwechslung [21].

Ein Anstieg der Anzahl von Menschen über 60 Jahren weltweit ist zu erwarten [22]. Basierend darauf finden substanzielle Veränderungen in der sozialen und familiären Struktur aller Personen, besonders aber bei älteren Menschen, statt [9, 19, 23]. Mit zunehmendem Alter kommt es zu vermehrter Pflegebedürftigkeit und Inanspruchnahme stationärer Pflegeeinrichtungen [24]. Das Setting Pflegeheim steht für die Absicherung des multidimensionalen Pflegebedarfs, der auch die umfassende Berücksichtigung sozialer und emotionaler Verhältnisse verlangt [25]. Dies ist allerdings erschwert durch einen Mangel an qualifiziertem Pflegefachpersonal [26]. Pflege wird aufgrund verschiedener Faktoren wie historische Entwicklung, mangelnde Edukation oder hierarchischer Aufbau des Gesundheitswesens zu oft auf Waschen, Ankleiden, Verbandwechsel oder Inkontinenzversorgung etc. reduziert, jedoch sollte die multidimensionale Pflege durch die Anpassung von Wohn‑, Betreuungs- und Pflegekonzepten in den Fokus rücken. Das Thema Einsamkeit soll besonderes aus pflegewissenschaftlicher Sicht thematisiert werden, da Pflegepersonen, gerade im Alters- und Pflegeheim, als erste Ansprechpersonen der Bewohner*innen eine große Rolle im Rahmen einer umfassenden Betreuung einnehmen.

## Ziel und Fragestellungen

Das Ziel dieser Arbeit ist es, das Erleben von Einsamkeit aus der subjektiven Perspektive der Bewohner*innen in der stationären Langzeitpflege zu beschreiben und Interventionen aufzuzeigen, die von Pflegepersonen eingesetzt werden können, um die Einsamkeit zu verringern. Es ergeben sich folgende Forschungsfragen: (1) Wie erleben ältere Bewohner*innen Einsamkeit im Setting der stationären Langzeitpflege? (2) Welche psychosozialen und spirituellen Unterstützungsmaßnahmen können von Pflegepersonen der stationären Langzeitpflege eingesetzt werden, um die Einsamkeit bei älteren Bewohner*innen zu verringern? Die Studie bezieht sich auf den europäischen Raum und das Setting stationäre Langzeitpflege, weil die hohe Anzahl an Pflegebedürftigen zu einer erhöhten Inanspruchnahme stationärer Pflegeeinrichtungen führt [27]. Gekoppelt an den Mangel an Pflegefachpersonal, verlangt dieses Problem wissensbasierte institutionelle Umsetzungen sowie landesübergreifende Maßnahmen [26].

## Methode

Zur Beantwortung der Forschungsfragen wurden eine systematische Literaturrecherche und -bewertung durchgeführt.

## Literaturrecherche

In der ersten Phase dieser Literaturarbeit wurde eine orientierende Recherche durchgeführt, die in der zweiten Phase durch eine regelgeleitete und systematische Recherche ergänzt wurde. Die Literaturauswahl wurde aufgrund zuvor definierter Einschluss- und Ausschlusskriterien (Zusatzmaterial online: Tab. 1) getroffen [28]. Die Medical Subject Headings (MeSH-Terms) wurden angewandt und durch Boole-Operatoren verknüpft (Zusatzmaterial online: Tab. 2). Die dritte Phase gründete auf der kritischen Analyse der Literatur [10, 29]. Die systematische Recherche wurde von Mai bis Juli 2019 durchgeführt. Hierfür wurden die Datenbanken *PubMed, CINAHL* und *LIVIVO* gesichtet. Unter Anwendung der „Berrypicking“-Methode konnte weitere Literatur identifiziert werden. Die Journals *BMC Geriatrics, Geriatric Nursing, Spiritual Care* und *Pflege* (ab 01.01.2009) wurden ebenfalls für die Recherche herangezogen. Insgesamt verblieben nach dem Ausschließen der Duplikate und der Volltextlesung 10 Studien. Die Analyse und kritische Würdigung erfolgten anhand der Beurteilungsbogen nach Behrens und Langer [29].

## Ergebnisse

Die Ergebnisse werden nachfolgend getrennt für beide Fragestellungen dargestellt.

## Erleben von Einsamkeit im Pflegeheim

Einsamkeitsgefühle im Pflegeheim sind sehr prominente Gefühle, jedoch sind Forschungsergebnisse über das Erleben der Einsamkeit von Bewohner*innen rar [11]. Paque et al. führten eine Studie in 3 flämischen Pflegeheimen bei Bewohner*innen ohne Demenzdiagnose und mit einem Mini Mental State Examination (MMSE) Score > 18 durch. Die Bewohner*innen wurden allgemein gefragt, mit welchen Gefühlen sie momentan Schwierigkeiten hätten. Spontan wurde selten die Einsamkeit primär erwähnt [11]. Je nach erhaltener Antwort wurde der Fokus im Interview auf die Einsamkeit gelenkt. Die Beschreibung der Einsamkeit divergierte vom Alleinsein über Langeweile bis hin zum Gefühl, sich nicht zu Hause zu fühlen [11]. Im Rahmen der Tiefeninterviews äußerte ein Bewohner, dass es ihm schwerfalle, über Einsamkeit zu sprechen, am ehesten könne er dies mit einem Gefühl des Unbeachtetseins oder der Ausgeschlossenheit beschreiben [11]. Die Untersuchung von Hauge und Kirkevold über einsame und nichteinsame ältere Menschen in institutionellen und kommunalen Settings zeigte, dass alle 30 interviewten Personen Schwierigkeiten damit hatten, Einsamkeit zu beschreiben [12]. Aussagen, die von Interviewten getroffen wurden, bezogen sich auf den Umstand, nicht besucht zu werden [12]. Es wurde festgestellt, dass sich nichteinsame Menschen negativ und kritisch gegenüber einsamen Menschen äußerten. Die Autor*innen führen den Begriff der Stigmatisierung ein, mit der Begründung, dass eine allgemeine negative Einstellung gegenüber einsamen Menschen in der westlichen Gesellschaft vorhanden sei [12]. Eine weitere Studie, durchgeführt mit 10 Teilnehmer*innen in Irland, zeigte, dass Einsamkeitsgefühle bei Pflegeheimbewohner*innen geäußert wurden, die sich der Langzeitpflegeeinrichtung nicht zugehörig fühlten [5]. Vier von 10 Personen gaben an, dass mit dem Umzug in das Pflegeheim ein Abgeschnittensein von der Außenwelt stattgefunden habe. Vor allem Personen mit einem aktiven Gemeinschaftsleben litten nach dem Einzug in die Langzeitpflegeeinrichtung unter der Einsamkeit [5]. Weitere Untersuchungen bestätigen diese Prozesse, die zu belastenden Einsamkeitsgefühlen und Problemen beim Knüpfen von Kontakten nach dem Einzug in die stationäre geriatrische Langzeitpflegeeinrichtung führen [12]. Bewohner*innen äußerten, dass auch aufgrund des physischen Zustandes und des dadurch entstandenen Verlustes der Selbstständigkeit eine Teilnahme an sozialen Aktivitäten nicht mehr möglich war. Wie Studien zeigen, ist die physische Konstitution ein wichtiger Faktor in der Entstehung und Wahrnehmung der Einsamkeit [12, 13].

Häufig ist das Einsamkeitserleben an einen schmerzlichen Verlust von oder Mangel an Freunden und Familienmitgliedern gekoppelt [5, 11–14]. In einer weiteren qualitativen Untersuchung wurden 12 depressive Pflegeheimbewohner*innen befragt [14]. Viele der Bewohner*innen erlitten schmerzliche Verluste naher Angehöriger. Bezogen auf das Personal äußerte der Großteil der Befragten, dass sie gern kommunizieren möchten, jedoch das Personal zu beschäftigt und überlastet wirke [14]. Aktives Zuhören, einfühlsames und wertschätzendes Vorgehen können zu einer Verringerung der Einsamkeit führen [5, 6]. Wiesen soziale Beziehungen eine hohe Qualität auf und wurde das Bedürfnis der Bewohner*innen nach bedeutungsvollen sozialen Kontakten befriedigt, verringerte sich das Einsamkeitserleben. Die Quantität an sozialen Kontakten und die Treffen mit anderen Bewohner*innen sowie der Kontakt mit Pflegepersonen oder anderen Gesundheitsberufen wurden als weniger bedeutungsvoll eingestuft [5, 11].

Bei der Befragung in einer irischen Langzeitpflegeinrichtung wurden Bewohner*innen, die zwischen 2 und 8 Jahre in der Institution wohnten, befragt, und niemand äußerte, dass eine bedeutungsvolle oder sinnhafte Beziehung zu anderen Menschen aufgebaut wurde [5]. Eine Verbindung mit der Außenwelt führte jedoch zu mehr sozialer Eingebundenheit und weniger Einsamkeit. Der Kontakt über diverse Medien wie Fernsehen oder Zeitung verhalf den Bewohner*innen nicht nur zu weniger Einsamkeit, sondern zu einem Gefühl der Zugehörigkeit [5]. Hierzu finden sich unterschiedliche Ansichten, denn auch die neuen Medien führen zu dem Eindruck, nicht mehr mit der Gesellschaft mithalten zu können [12]. Auch der Kontakt mit Bewohner*innen mit kognitiven Einschränkungen ist problematisch. Kognitive Defizite anderer Bewohner*innen führten zum vermehrten Rückzug, und die kognitiv uneingeschränkten Bewohner*innen beschrieben sich als anders, nicht zugehörig [5].

Die Erforschung des Phänomens „Lebensqualität“ in Verbindung mit Einsamkeit bei 250 Bewohner*innen in Polen ergab, dass ältere (76+) weibliche Teilnehmerinnen sich häufiger und intensiver einsam fühlten als männliche Teilnehmer. Hierfür wurden die De Jong Gierveld Loneliness Scale (DJGLS) [15] und der WHOQOL-BREF-Fragebogen [30] eingesetzt [13]. Eine signifikante Korrelation (*p* < 0,041) besteht zwischen nichtzufriedenstellenden oder unzureichenden (ρ = −0,362 *p* < 0,001) Beziehungen zur eigenen Familie und erhöhtem Einsamkeitserleben [13].

## Maßnahmen zur Verringerung der Einsamkeit

Kuru Alıcı et al. untersuchten die Effekte einer Lachtherapie in 2 Pflegeheimen in der Türkei. Die Bewohner*innen wurden nicht-randomisiert einer Interventions- und Kontrollgruppe zugewiesen. Eine Ermittlung der Stichprobengröße erfolgte mittels Power-Analyse. Insgesamt wurden 50 Proband*innen eingeschlossen. Die Interventionsgruppe erhielt 2 Tage/Woche über 5 Wochen für ca. 35–40 min Lachyoga-Einheiten. Die Kontrollgruppe erhielt keinerlei Intervention. Die Werte der DJGLS [15] und der Turkish Death Anxiety Scale (TDAS) [31] wurden während des ersten und nach dem letzten Interview erhoben [32]. Vor der Intervention wurde keine signifikante Differenz (*p* = 0,209) zwischen Interventions- ($\bar{x}$ = 17,95 ± 2,704) und Kontrollgruppe ($\bar{x}$ = 16,77 ± 3,510) festgestellt. Nach der Intervention zeigte sich ein signifikanter Unterschied in den Werten der DJGLS zwischen der Interventionsgruppe ($\bar{x}$ = 7,15 ± 1,755; *p* = 0,001) und der Kontrollgruppe ($\bar{x}$ = 15,63 ± 5,027; *p* = 0,001). Einsamkeit in der Interventionsgruppe wurde signifikant verringert (t = 7,237; *p* = 0,001) [32]. Insgesamt konnte kein Unterschied vor und nach der Anwendung in der TDAS festgestellt werden [32].

Die Anwendung von technologischen Interventionen beherbergt ebenfalls Potenzial. In der Studie von Zamir et al. (2018) wurde das Ziel gesetzt, primär die Durchführbarkeit und Anwendbarkeit von Skype on Wheels bei älteren Menschen in „care settings“ festzustellen. Ein weiteres Ziel bestand darin, potenzielle Designänderungen oder Alternativen zu ergründen und Barrieren, Möglichkeiten und Vorteile von Videoanrufen der Bewohner*innen, Angehörigen und des Personals zu identifizieren. In die Studie wurden 8 Pflegeheimbewohner*innen, 10 Patient*innen und 9 Angehörige aus dem Vereinigten Königreich eingeschlossen [33]. Die qualitative Analyse ergab, dass dies eine Möglichkeit der Reduktion von Einsamkeit und sozialer Isolation darstellt. Mangelnder Einsatz aufgrund von zeitlichen Ressourcen des Personals erschwert jedoch die Applikation dieser Maßnahme. Auch existierten Probleme bei der Durchführbarkeit und Akzeptanz der Bewohner*innen und des Personals. Insgesamt nutzte die Hälfte der Proband*innen das Gerät ein- bis 2‑mal/Monat [33].

In der Studie von Vrbanac et al. (2013) wurde durch tiergestützte Therapie versucht zu ermitteln, ob diese Therapieform die Wahrnehmung der Einsamkeit bei Pflegeheimbewohner*innen reduziert [34]. In dieser Studie aus Kroatien wurden die UCLA Scale of Loneliness [35] (vor Beginn und nach 6 Monaten) sowie demografische Merkmale der Proband*innen erhoben. Es wurden Befragungen zum Alltag im Pflegeheim (Kontakt zu anderen, Aktivitäten), zur Einstellung zu Tieren und zu Erfahrungen mit tiergestützter Therapie durchgeführt und zudem wurden Daten der Observation während der Interaktion gesammelt. Insgesamt wurden 21 Pflegeheimbewohner*innen in die Studie einbezogen. Der t‑Test für abhängige Stichproben wurde berechnet, und ein Signifikanzniveau (*p* < 0,01) wurde festgelegt [34]. Die Intervention zeigte eine statistisch signifikante Verbesserung nach der Therapie auf der UCLA Scale of Loneliness (t = 4,261; df = 20; *p* = 0,003). Die höchste Verbesserung erfolgte beim Statement: „I lack company“ (t = 6,821; df = 20; *p* = 0,000). Die häufigsten Interaktionen waren Sprechen, Kuscheln und Ausdruck von Freude durch Lächeln. 96 % (*n* = 20) gaben an, dass ihre Stimmung dadurch verbessert wurde [34].

Die letzte gefundene Maßnahme wurde von Downey beschrieben und in dem Vereinigten Königreich durchgeführt [36]. Mit Unterstützung des Verwandten- und Bewohnerverbandes wurde eine einjährige Pilotstudie gestartet, mit dem Ziel, ein Modell des freiwilligen Helfens in Pflegeheimen zu erschaffen. An der Studie nahmen 4 Student*innen und 9 Pflegeheimbewohner*innen teil [36]. Über eine Dauer von insgesamt 8 Wochen fanden Besuche einmal/Woche und eine Stunde pro zugeteilter/zugeteiltem Bewohner*in statt. Die Datenerhebung erfolgte qualitativ durch Observationen, Tagebücher der Student*innen, Feedbackrunden in Meetings und Evaluationsbogen am Studienende. Die Pilotstudie erzeugte positives Feedback von Bewohner*innen. Die Bewohner*innen gaben an, dass sie sich durch die Besuche und Gespräche wertgeschätzt fühlten. Darüber hinaus wurde die eingeplante Zeit, in der jemand für sie da sei, von allen beteiligten Personen positiv erwähnt. Laut Autorin bestand mehr Zeit für persönliche Gespräche, den Aufbau eines sozialen Netzes und die Arbeit an der Biografie von Bewohner*innen. Besonderes durch Feedbackrunden während der Besprechungen wurde das Verständnis für Einsamkeit und soziale Isolation verbessert [36].

## Diskussion

Die subjektive Betrachtung des Phänomens Einsamkeit bedarf der Einbeziehung vieler Aspekte [5, 11–14]. Oft bestehen Schwierigkeiten, über die eigene Einsamkeit zu sprechen und diese zu artikulieren. In Interviews zu dem Thema variierten Ausprägungen der Einsamkeit von Alleinsein über Langeweile bis zu dem Gefühl, sich nicht zu Hause zu fühlen. Die Einsamkeit wird nicht uneingeschränkt als negativ von Bewohner*innen aufgefasst. Auch positive Einsamkeit wird empfunden, obwohl negative, emotionale und soziale Einsamkeit dominiert [19]. In den einzelnen Publikationen variierten die Definitionen, Erörterungen oder Erläuterungen bezüglich Einsamkeit, negativer Einsamkeit, sozialer Isolation und Einsamkeit als existenziellem Zustand [37]. Diese Variation und die heterogenen Forschungsdesigns verursachen eine begriffliche Intransparenz und erschweren eine systematische Analyse der Daten [28].

Am Beginn des Einsamkeitserlebens stehen häufig schmerzliche Verluste von Angehörigen, Freunden oder anderen im jeweiligen Leben bedeutsamen Menschen. Diese Entwicklungen sorgen bei institutionell betreuten Menschen für weitere schmerzliche Gefühle von Einsamkeit [13]. Der Einzug in eine Einrichtung der stationären geriatrischen Langzeitpflege führt bei einigen Bewohner*innen zu einer Isolation von der Außenwelt [5]. Fehlende Autonomie, eingeschränkte Partizipation und Heimweh waren damit assoziierte Gefühle. Bewohner*innen ohne oder mit geringen kognitiven Defiziten gaben an, dass sie den Kontakt mit Personen mit demenziellen Erkrankungen mieden, da diese alles vergessen würden und eine sinnvolle Kommunikation nicht mehr möglich wäre [5]. Das Phänomen der Einsamkeit inmitten der Menge manifestiert sich in diesen Aussagen [14]. Aktives Zuhören, die Einstellung des Pflegefachpersonals gegenüber Einsamkeit und das aktive Handeln des Personals werden als hilfreich eingestuft [5].

Maßnahmen zur Reduktion von Einsamkeit reichen über die Anwendung von Lachtherapie, tiergestützter Therapie bis zu (hoch)technologischen Maßnahmen [9]. Der Einsatz von Lachtherapie zeigte, dass diese zu einer Verringerung der Einsamkeit führen kann und eine sinnvolle Gruppenaktivität zur Festigung sozialer Netzwerke darstellt [32]. Die tiergestützte Therapie konnte ebenfalls diesen Effekt erzielen [9]. Die High-Tech-Interventionen zeichnen sich durch Ambivalenz aus. Die Überbrückung von Distanzen kann eine bereichernde Methode darstellen, doch Untersuchungen zur Anwendbarkeit und zur Edukation des Pflegefachpersonals und der Bewohner*innen stehen noch aus [33, 38]. Der Einsatz von Freiwilligen wurde auf kommunaler Ebene bereits getestet, umgesetzt und könnte eine mögliche Intervention sein [39].

Neben diesen innovativen und z. T. hochtechnologischen Maßnahmen erwiesen sich grundlegende zwischenmenschliche Prozesse als besonders bedeutend. Hierbei wurden folgende Aspekte als zentral beschrieben: qualitativ hochwertige Beziehungen/ein erfülltes Beziehungsleben ermöglichen [5, 11], aktives Zuhören sowie einfühlsame und wertschätzende Kommunikation [5]. Dies sind Maßnahmen, welche von Pflegefachkräften, wenn diese über genügend Ressourcen und eine gute Ausbildung verfügen, im Pflegealltag gut integriert werden können.

## Limitationen

Die Stärke dieser Arbeit liegt in der erstmaligen umfassenderen Betrachtung des subjektiven Erlebens der Einsamkeit aus der Perspektive von Bewohner*innen in Pflegeheimen. Die Autor*innen beziehen sich bei der Darstellung des Erlebens von Einsamkeit und bei der Präsentation der Maßnahmen auf wissenschaftliche Studien. Durch die Recherche mit dem Fokus auf Pflegeheime wurde nur ein Ausschnitt der aktuellen Forschung dargestellt. Ein Großteil der Studien über Einsamkeit inkludiert das eigene Heim, die mobile Hauskrankenpflege sowie „community health nursing“. In ihrer Erklärung zum gesunden Altern betont die WHO gemeinsam mit dem International Council of Nurses (ICN) die Schlüsselrolle von Pflegefachpersonen in der integrierten personenzentrierten Gesundheitsversorgung für ältere Menschen. Die Betreuung in Communities soll durch die Vorbeugung von Krankheiten, durch Fürsorge und Behandlung sowie durch ein gutes Symptommanagement im Netzwerk mit psychologischer, sozialer und spiritueller Unterstützung bewältigt werden [22, 40]. Eine weitere Einschränkung ergibt sich durch den Fokus auf den europäischen Raum. Die Mehrzahl der Studien zu diesem Thema wurde in nordamerikanischen Regionen und auf dem asiatischen Kontinent durchgeführt. Die heterogene Studienlage ist anzumerken, da diese auch die Analyse und eine Ergebnissynthese erschwert.

## Schlussfolgerungen und Ausblick

Das Wohnen in einem Pflegeheim bringt bestimmte strukturelle Umstände mit sich, die das Erleben von Einsamkeit verstärken können. Diese Emotionen, die neben Einsamkeit noch weitere Empfindungen umfassen, werden oft nicht geäußert, da sich die Verbalisierung schwierig gestaltet und einsame Personen zudem gesellschaftlicher Stigmatisierung ausgesetzt sind [12]. Der aktuelle Forschungsstand zeigt jedoch auf, dass wirksame Maßnahmen zur Reduktion von Einsamkeit im Pflegeheim eingesetzt werden können. Als zentral und am wirkungsvollsten haben sich jene Interventionen erwiesen, welche die spirituelle Ebene des Beziehungsaufbaus und Vertrauen fokussieren. Sowohl Pflegepersonen als auch Angehörige können darauf achten, dass qualitativ hochwertige Beziehungen gepflegt werden. Dies erfüllt das zentrale Bedürfnis nach einfühlsamer und wertschätzender Kommunikation sowie nach aktivem Zuhören [5, 11]. Bestimmte Medien und Technologien können auch zu einer Verringerung des Einsamkeitserlebens führen. Obwohl diese Maßnahmen von vielen Bewohner*innen positiv bewertet wurden, zeigten sich auch Reaktionen wie Skepsis oder Ablehnung [12]. Aus diesem Grund muss die Angemessenheit der Maßnahmen immer individuell abgewogen werden.

## Fazit

Einsamkeit als genuin menschliches Phänomen betrifft viele Bewohner*innen von Pflegeheimen. Die Studien zeigen, dass das Erleben von Einsamkeit schwer zu äußern ist, und evtl. wird auch bewusst darüber geschwiegen. Häufig ist das Einsamkeitserleben an einen schmerzlichen Verlust von oder Mangel an Freunden und Familienmitgliedern gekoppelt. Auch eine schlechte physische Konstitution kann zu Autonomieverlust und in weiterer Folge zu Einsamkeit führen. Der Einzug in ein Pflegeheim bedeutet oft den Verlust von sozialen Kontakten. Selten werden im Pflegeheim bedeutungsvolle oder sinnhafte Beziehungen zu anderen Bewohner*innen aufgebaut. Zeitmangel des Personals und Kontakt zu kognitiv eingeschränkten Personen können das Einsamkeitsgefühl verstärken. Besonders ältere weibliche Bewohnerinnen fühlen sich öfter und intensiver einsam als männliche Bewohner von Pflegeheimen.

## Supplementary Information





