# Supplementary material for: Loneliness in nursing homes—Experience and measures for amelioration: A literature review
Source: Z Gerontol Geriatr. 2021 Apr 15;55(1):5–10. [Article in German] doi: 10.1007/s00391-021-01881-z (PMC8789688; doi:10.1007/s00391-021-01881-z)
Supplement: Supplementary file 1 [file 391_2021_1881_MOESM1_ESM.docx]

|  | Einschlusskriterien | Ausschlusskriterien |
| --- | --- | --- |
| Phänomen | Erleben der Einsamkeit;  Psychosoziale und spirituelle Maßnahmen zur Verringerung von Einsamkeit;  Definition bzw. Erörterung von Einsamkeit | Medikamentöse Maßnahmen zur Verringerung von Einsamkeit, Maßnahmen ohne Fokus auf Einsamkeit |
| Population | Ältere (>65 Jahren) Bewohner*innen eines Pflegeheimes/Stationäre geriatrische Langzeitpflege | Bewohner*innen anderer Einrichtungen (Demenzstation, Palliativstation) |
| Setting/Versorgungsform | Pflegeheim, Stationäre Langzeitpflege | Akutstationäre oder ambulante Betreuung, Mobile Pflege, Hauskrankenpflege, Hospiz |
| Publikationsart | Primärstudien, Reviews, Fachbeiträge | Nicht-wissenschaftliche Literatur |
| Sprache | Deutsch, Englisch | Alle anderen Sprachen |
| Geographischer Raum | Europa | Studien aus anderen Ländern |
| Publikationszeitraum | 2009 – 2019 (Ausnahme Priorität für Arbeit) | Literatur vor 2009 |
| Journal | Peer-review, Abstract verfügbar | Ohne Abstract, kein Peer-Review |

Tabelle 1: Einschluss- und Ausschlusskriterien (Eigene Darstellung, 2019)
