# Supplementary material for: Loneliness in nursing homes—Experience and measures for amelioration: A literature review
Source: Z Gerontol Geriatr. 2021 Apr 15;55(1):5–10. [Article in German] doi: 10.1007/s00391-021-01881-z (PMC8789688; doi:10.1007/s00391-021-01881-z)
Supplement: Supplementary file 2 [file 391_2021_1881_MOESM2_ESM.docx]

|  | Deutsche Suchbegriffe | Englische Suchbegriffe |
| --- | --- | --- |
| Komponente 1:  Erleben | Erleben, Erfahrung, Einstellung, Wahrnehmung, psychosoziale Faktoren | experience, perception, attitude, psychosocial factors |
| Komponente 2:  Bewohner*innen | Pflegeheimbewohner*innen, Heimbewohner*innen | nursing home patients, nursing home residents, elderly people |
| Komponente 3:  Setting | Pflegeheim, Altenpflegeheim, Geriatrische Pflege, Langzeitpflege, Gerontologie | nursing home, geriatric nursing, long-term care, long-term facilities, gerontologic care, elderly care |
| Komponente 4:  Einsamkeit | Einsamkeit, Soziale Isolation, Isolation, Alleinsein | loneliness, social isolation, isolation |
| Komponente 5:  Maßnahmen | Psychosoziale Maßnahmen/Unterstützung, Soziale Unterstützung Spirituelle Maßnahmen/Unterstützung,  Pflegeintervention, Spiritualität, Unterstützung, Interventionen | psychosocial support/intervention/system, social support, spiritual support/intervention/system, spiritual care, spirituality, support, intervention |

Tabelle 2: Deutsche und englische Suchbegriffe (Eigene Darstellung, 2019)
